# Is There Evidence of Poorer Birth Outcomes for Mothers and Babies When the Most Senior Obstetrician Is Not On Site?

**DOI:** 10.1371/journal.pmed.1002001

**Published:** 2016-04-19

**Authors:** Jenny E. Myers, Edward D. Johnstone

**Affiliations:** Maternal and Fetal Health Research Centre, University of Manchester, Manchester, United Kingdom

## Abstract

In this Perspective on the study by Hannah Knight and colleagues, Jenny Myers and Edward Johnstone consider the implications of negative findings in a variable setting in which adverse events are rare.

The idea of medical care provided by on-site senior staff 24 hours per day is inherently attractive to both users and providers of health services. Intuitively, having the most senior doctor on site at all times would be associated with improvements in care delivery. Evidence has suggested that mortality rates are significantly higher for adults admitted during the weekends [1], and studies from the United Kingdom and other countries have suggested higher perinatal mortality rates during these times [2,3]. As a result of both perception and evidence, the UK government is making efforts to change working patterns for all grades of doctors, including consultants (specialists who have completed their training), to reduce the variation in the level of experience and training of medical staff providing front-line care during a 24-hour period and throughout the week [4].

Against this backdrop, the study in this week’s *PLOS Medicine* by Hannah Knight and colleagues is timely [5]. The authors present a UK-based study examining over 87,000 births from 19 maternity units, with varying numbers of hours of consultant presence throughout the weekday and at the weekend. The authors did not find evidence that short-term neonatal outcomes were significantly worse when the baby was born “out of hours,” when a consultant was not routinely on site in the hospital, but they did find marginally lower rates of intrapartum Caesarean section and operative vaginal delivery.

This is the largest contemporary UK study using hospital-level statistics in conjunction with information regarding consultant and junior doctor working patterns. One of the main strengths of the study is that it included both large university hospitals and smaller secondary care institutions, the laudable intention being to provide generalizable information pertinent to the whole of the UK and other countries with similar models of maternity care. In the units included, on-site consultant presence ranged from 51–106 hours per week. The most noteworthy finding of the study was the absence of any statistically significant increase in the frequency of infants born with low Apgar scores or signs of hypoxia (umbilical cord pH <7.1), or in the frequency of admissions to the neonatal unit. Whilst limited, these metrics are commonly used to evaluate intrapartum asphyxia and therefore, indirectly, the quality of intrapartum care provided. Given concerns from previous reports that the lack of a trained specialist has the potential to negatively impact on the quality of intrapartum care [6], it might be hypothesized that the frequency of infants born in poor condition would be higher “out of hours.” The impact of the authors’ findings in this study is therefore highly relevant for care providers and policy makers.

The authors are careful not to overstate their findings and appropriately discuss the limitations of the study. In particular, information regarding consultant presence was estimated from consultant rotas, and information about actual consultant presence was not available. Furthermore, data on other aspects of the organization of maternity care in the units studied, including the training levels of on-site medical staff, were not collected. It could also be supposed that the units that volunteered to participate and had robust electronic data capture systems might have more sophisticated clinical governance systems in place. It is therefore possible that the quality of intrapartum care provided in these units is higher than the average across the UK.

Despite this being a large study, the rarity of serious adverse neonatal outcomes means that there is a potential for type 2 error. The study was more than adequately powered to detect a 10% change in the rate of admissions to the neonatal intensive care unit (5.93% out of hours versus 6.73% in hours; adjusted odds ratio [OR] 0.99, 95% confidence interval [CI] 0.93–1.06). However, the study was underpowered to detect a similar magnitude of change in proportions of babies with low Apgar scores or fetal acidemia at birth. In terms of fetal acidemia, there was a nonsignificant increase of 12% (0.94% out of hours versus 0.82% in hours, adjusted OR 1.12, 95% CI 0.96–1.31). This size of difference would equate to approximately 467 additional babies born out of hours with a low cord pH, given the 700,000 births expected per year in the UK, although this risk estimate has a wide confidence interval.

It is also possible and plausible that the higher Caesarean section and operative delivery rate in the “in hours” deliveries could account for the lower prevalence of fetal acidemia and low Apgar scores in this group; this could result from an increase in appropriately timed interventions by more senior clinicians in the context of suspected fetal distress. The authors have, quite correctly, not made this claim as the study was not powered or designed to address this question.

Despite the inclusion of a broad mix of units across the UK in Knight and colleagues’ study, application of the findings to individual hospital settings, even within the UK, is difficult. The necessity for consultant cover is likely to differ significantly across different regions depending on the level of training of the on-site junior doctor cover, the complexity of the case mix, and the annual delivery rate. There will also be a broad disparity in the level of experience, along with the frequency of exposure to the labour/delivery unit, of the consultant staff in different maternity units. Some rotas will include generalists, who continue to practice all aspects of obstetrics and gynaecology, with others made up of specialist obstetricians whose practice is focused on high-risk obstetrics. Our own institution (which was not included in Knight and colleagues’ study) now has 24-hour, seven-days-a-week, on-site consultant cover, but has struggled to maintain continuity of staffing due to the large number of consultants required to staff such rotas. This often results in reliance on nonpermanent staff. Although there was no evidence of staff turnover within the study sites in Knight and colleagues’ study, it is possible that sites with higher levels of cover are also experiencing this problem.

Outside the UK, many other countries maintain an on-call rota system with the senior member of the team away from the hospital out of hours. This contrasts to the on-site presence of senior staff more commonly found in North America. To our knowledge, there is no evidence of the superiority of one system over another; there is, however, considerable disparity in the cost of these different models of maternity care. Whilst debate rages in the UK over the provision of out of hours obstetric care and secondary care in general [4], there is actually very little evidence available, particularly on birth outcomes. A policy that unreservedly supports 24-hour on-site consultant cover, resulting in enormous additional resource use and complex service reorganization, should therefore be cautioned against, given the evidence presented by Knight and colleagues.
